# The Genomic Characteristics of an Arthritis-Causing *Salmonella* pullorum

**DOI:** 10.3390/microorganisms11122986

**Published:** 2023-12-14

**Authors:** Zhiyuan Lu, Jiaqi Huang, Peiyong Li, Mengze Song, Ben Liu, Wenli Tang, Shuhong Sun

**Affiliations:** 1 Department of Preventive Veterinary Medicine, College of Animal Science and Technology, Shandong Agricultural University, Taian 271018, China; 2022110423@sdau.edu.cn (Z.L.); 2020110516@sdau.edu.cn (J.H.); 15163823626@163.com (P.L.); songmz@sdau.edu.cn (M.S.); liuben117@163.com (B.L.); 2 Shandong Center for Quality Control of Feed and Veterinary Drug, Shandong Provincial Key Laboratory of Quality Safty Monitoring and Risk Assessment for Animal Products, Jinan 250100, China

**Keywords:** *Salmonella* pullorum, arthritis, whole-genome sequencing, comparative genomics

## Abstract

*Salmonella enterica* subsp. *enterica* serovar Gallinarum biovar pullorum (*Salmonella* pullorum) is an avian-specific pathogen that has caused considerable economic losses to the poultry industry. High endemicity, poor implementation of hygiene measures, and lack of effective vaccines hinder the prevention and control of this disease in intensively maintained poultry flocks. In recent years, the incidence of arthritis in chicks caused by *Salmonella* pullorum infection has increased. In this study, four *Salmonella* pullorum strains were identified from the livers, spleens, and joint fluids of Qingjiaoma chicken breeders with arthritis clinical signs, and an arthritis model of chicks was successfully established using SP206-2. Whole genome sequencing of the SP206-2 strain showed that the genome was 4,730,579 bp, 52.16% GC content, and contained 5007 genes, including 4729 protein-coding regions. The genomic analysis of four arthritis-causing isolates and three diarrhea-causing isolates showed that the genome of arthritis-causing isolates was subject to nonsynonymous mutations, shift mutations, and gene copy deletions. An SNP phylogenetic tree analysis showed that arthritis-causing isolates are located in a different evolutionary branch from diarrhea-causing isolates. Further differential genes analysis showed that the genome of arthritis-causing isolates had missense mutations in genes related to substance metabolism and substance transport, as a result of adaptive evolution.

## 1. Introduction

*Salmonella* is one of the most important pathogens that threatens the poultry sector worldwide right now [1]. Most of these serotypes can infect different hosts, like *Salmonella* enteritidis (*S*. enteritidis) and *Salmonella* typhimurium (*S*. typhimurium). *Salmonella* pullorum (*S*. pullorum) and *Salmonella* gallinarium (Fowl typhoid) are the most pathogenic serovars in avian species leading to systemic infection [2]. *S*. pullorum can cause pullorum disease through both horizontal and vertical transmission, and it primarily affects chickens and turkeys [2]. Different breeds of chickens have different susceptibilities to *S*. pullorum; in general, light chickens are more tolerant than heavy chickens [3]. In addition, the age of chickens is also associated with susceptibility to *S*. pullorum: chicks and poults less than 2–3 weeks old are vulnerable to attack from *S*. pullorum, with acute septicemia and high mortality [4]. Although adult chickens can often be asymptomatic carriers of the bacteria throughout their lives, they can also show clinical signs such as weight loss, decreased fertility and hatchability, diarrhea, and reproductive tract abnormalities [5]. In addition, *S*. pullorum can persist for many months in the spleen and reproductive tract, resulting in its vertical transmission to eggs and progeny [6]. Other signs associated with *S.* pullorum infection, such as purulent arthritis and joint lesions, have occasionally been described [7]. Although *S*. pullorum has been under control and not a primary concern in developed countries benefiting from the great process of intensive poultry industry and the implementation of extensive eradication programs, *S.* pullorum is still prevalent in China, due to the diversity of the poultry industry and breeding patterns. This severely constrains the healthy development of the breeding chicken industry [8].

Arthritis commonly manifests in lameness, swollen tarsal joints, and prolonged lying in chickens, particularly in broilers. Restricted movement due to tarsal joint disease results in decreased feed intake, weight loss, and lower egg production. Primary etiological agents associated with arthritis include avian *reovirus*, *Mycoplasma synoviae*, *Staphylococcus*, *Escherichia coli* (*E. coli*) [9,10,11,12], *Campylobacter*, *Enterococcus cecorum*, and *Enterococcus faecalis* [13,14,15]. Partial serotypes of *Salmonella* can also induce arthritis, mainly those containing *S.* enteritidis and *S.* typhimurium, as reported [16,17]. In recent years, triggered by *S*. pullorum, arthritis is rising in China, which is becoming a new epidemic characteristic of *S.* pullorum.

With the rapid development of whole-genome sequencing (WGS) technology, several strains of *S.* pullorum have been sequenced and annotated with a whole genome. The availability of more genome sequences has led to the discovery of additional genes, and has fueled the new field of comparative genomics. Comparative genome analysis provides a new means for studying the pathogenic mechanism and the genome evolution of *S.* pullorum, promoting the monitoring and tracing of *S*. pullorum, resistance analysis, and vaccine development through the comparative analysis of genes and gene families among representative species in a phylogeny and the construction of phylogenetic maps [18].

In a previous work, we isolated four strains of *S*. pullorum from the joint fluid and organs of native Chinese Qingjiaoma chicken breeds with swollen tarsal joints and cysts, and we reproduced the joint lesions in an experimental chick infection model. Our research showed that the hypervirulent arthritis-causing *S*. pullorum strain SP206-2 had greater negative impact on the growth performance of chicks compared to the white diarrhea-causing standard *S*. pullorum strain [19]. The current study analyzes the genomic features of arthritis-causing *S*. pullorum, and explores the differential expression genes with the white diarrhea-causing *S*. pullorum, using WGS as well as genomics. The results provide new insights into the evolution of *S*. pullorum and epidemiological surveillance of pullorum disease.

## 2. Materials and Methods

### 2.1. Bacterial Strain and Growth Conditions

Four arthritis-causing *S*. pullorum strains were isolated from the synovial fluid of swollen hock joint and heart in native Chinese Qingjiaoma chicken breeders with clinical signs of arthritis. *S*. pullorum strain SP-A100 was isolated from dead embryos of native Chinese blue-shell chickens. The standard strain of *S*. pullorum, CVCC526, was provided with the China Veterinary Culture Collection Center (Beijing, China). All *Salmonella* strains were stored at −70 °C. The strains were first streaked onto xylose lysine deoxycholate agar plates and then incubated at 37 °C for 24 h for subsequent tests.

### 2.2. Growth Curves

Fresh overnight cultures of SP206-2 and CVCC526 were diluted to a final concentration of 10^6^ CFU/mL in Luria-Bertani (LB) broth medium and M9 Minamal medium, respectively, and incubated continuously for 16 h at 37 °C, 220 rpm. The optical density (OD_600_) was measured using SpectraMax i3x microplate reader (Molecular Devices, Silicon Valley, CA, USA), at intervals of 2 h, for drawing of the growth curve.

### 2.3. Minimum Inhibitory Concentration (MIC) Assay

Drug susceptibility testing was performed according to the microbroth dilution method, recommended by the Clinical and Laboratory Standard Institute (CLSI) [20]. Single colonies were picked and incubated to the logarithmic phase at 37 °C, 200 rpm in Mueller–Hinton broth medium, then diluted to a final concentration of 10^5^ CFU/mL. The MIC of gentamycin (0.125 µg/mL~8 µg/mL), tetracycline (0.125 µg/mL~8 µg/mL), ciprofloxacin (0.03 µg/mL~2 µg/mL), and ampicillin (4 µg/mL~4096 µg/mL) against three strains of *S*. pullorum were detected in three parallel experiments in 96-well plates, using two-fold serial gradient dilutions and incubated at 35 °C for 16–20 h. The MIC is the lowest drug concentration in the pores that completely inhibits the growth of bacteria.

### 2.4. RNA Isolation and Quantitative Real-Time PCR (qPCR)

Total RNA was extracted using Trizol reagent (Invitrogen, California, USA), following the manufacturer’s protocol; this was followed by reverse transcription into cDNA using HiScript II Q RT SuperMix for qPCR (+gDNA wiper) (Vazyme, Nanjing, China). Primers were designed by the software Primer 5 and synthesized by Sangon Biotech Co., Ltd. (Shanghai, China). Then, PCR reactions were conducted using the primers listed in Table 1 with the following programs: 1 cycle at 95 °C for 30 s, 40 cycles at 95 °C for 5 s, and 60 °C for 5 s. Relative expression levels in arthritis-causing *S*. pullorum compared with diarrhea-causing *S*. pullorum were calculated using the 2^−ΔΔCt^ method, with 16 S rRNA as the reference gene [11].

### 2.5. Extracting and Sequencing Bacterial Genome Library Construction

The whole genome of bacterial was extracted using the Bacterial Genome Extraction Kit (Tiangen, Beijing, China). Then, the total amount of DNA was detected using the Quant-iT PicoGreen dsDNA Assay Kit (Thermofisher, Shanghai, China), and the DNA integrity was detected through 1% agarose gel electrophoresis. The qualified samples were sent to Novogene Co., Ltd. (Beijing, China) for WGS. DNA samples were randomly interrupted with an ultrasonic crushing apparatus to create fragments of approximately 350 bp in length. Subsequently, the entire Library preparation was completed with the NEBNext^®^Ultra™ DNA Library Prep Kitfor Illumina (NEB, Ipswich, MA, USA) kit through the steps of end repair, addition of A-tails, addition of sequencing adapters, purification, and PCR amplification. Illumina NovaSeq PE150 sequencing was performed with different libraries according to the effective concentration and the target amount of data.

### 2.6. Gene Prediction

Coding genes were predicted by Prodigal [21], tRNA by Aragorn [22], rRNA by RNAmmer [23], and miscRNA by Infernal [24]. Pseudogenes were screened from pseudogene candidate sequences of bacteria and archaea genomes in annotated Genbank files through Pseudofinder (https://github.com/filip-husnik/pseudofinder accessed on 16 November 2023). The genome circle was mapped using the genomic information obtained from the prediction. We used the R package circlize to map the genome circle and make the genome visualized [12].

### 2.7. Gene Function Annotation

In order to obtain comprehensive gene function information, gene function annotation was performed with nine major databases, including UniProt, KEGG, KEGG Pathway, GO, Pfam, COG, TIGERfams, RefSeq, and NR. The predicted gene sequences were compared with COG, KEGG, Swiss-Prot, Refseq, and other functional databases using BLAST+ in order to obtain gene functional annotation results. Hmmer software was used for functional annotation, based on the Pfam and TIGERFAM databases [13]. The amino acid sequences of the target species were aligned to the CARD database using the Resistance Gene Identifier [14]. Software was provided by the CARD database (RGI built-in blastp, default “evalue ≤ 1 × 10^−30^”) [15]. The drug resistance gene information was statistically annotated to the database according to the alignment results of RGI.

### 2.8. Genomic Variability Analysis of Arthritis-Causing S. pullorum

Genomic variability analysis was performed on the genomes of four sequenced arthritis-causing *S*. pullorum strains and the diarrhea-causing *S*. pullorum strains CVCC526, CVCC1795 (GCF_019990585.1), and SGSC25088 (GCA_018338655.1).

#### 2.8.1. ANI Analysis

FastANI (v1.33) software was used to analyze the Average Nucleotide Identity (ANI) of the genome and to draw the heat map of similarity [16]. The constructed genome map of SP206-2 was used as the reference sequence for genome variation analysis.

#### 2.8.2. Constructing the Reference Sequence Index File

The reference sequence index file for sequence alignment was generated using the “bwa index” parameter; the fai file required for subsequent alignment was generated using the “samtools(v1.14) faidx” parameter; and the “picard CreateSequenceDictionary” parameter generated the reference sequence index file for variant analysis.

#### 2.8.3. Sequence Alignment

The fastq documents of clean data obtained from quality control were compared to the reference co-gene group SP206-2 by means of “bwa(0.7.17) mem” parameters with “-t 64 -M -P -R “@RG\tID:$file\tSM:$file\tLB:$file\tPL:Illumina”,”. The resulting bam files were preprocessed and sorted using samtools alignment with the parameters “samtools view-bS” and “samtools sort”. The processing of multiple comparisons was performed by picard MarkDuplicates with “picard.jar MarkDuplicates REMOVE_DUPLICATES = falseMAX_FILE_HANDLES_FOR_READ_ENDS_MAP = 800”.

#### 2.8.4. Recalibration Base Quality Score (BQSR)

Analysis of genomic variant sites was performed using gatk [17]. First, Haplotype Caller software was used to process the mutation site with the parameter “gatk(v4.0)—java-options—Xmx8G HaplotypeCaller—R complete_genome.fasta—ERC GVCF—max-mnp-distance 0”. The gvcf files generated in this step were then merged using the CombineGVCFs software and then further analyzed the mutation loci.

#### 2.8.5. Quality Control and Filtering of Variant Sites

Single Nucleotide Polymorphism (SNP) and Insertion and Deletion (INDEL) sites were screened using SelectVariants software. SNP sites were screened using VariantFiltration, and the threshold was set to “QD < 2.0, QUAL < 30.0, SOR > 3.0, FS > 60.0, MQ < 40.0, MQRankSum < −12.5, ReadPosRankSum < −8.0”. The output of INDEL was performed by VariantsToTable.

#### 2.8.6. Phylogenetic Tree Construction

Distance analysis of SNP information was performed using VCF2Dis [18] software, and the output was a distance matrix in mat format. FastME (v2.0) [19] was used to input the SNP distance matrix file. Phylogenetic tree visualization was performed using the R language ggtree package or MEGA6.

### 2.9. Enrichment of Differential Genes

#### 2.9.1. Screening for Nonsynonymous Mutations

Perl script was used to re-predict the genomic coding sequence (CDS) after replacing the mutation sites, and blastp software was used to align the protein sequences before and after mutation at the mutation sites. After the identity results were ordered from smallest to largest, sequence results with <100% identity were screened for subsequent analysis.

#### 2.9.2. VCF Information Statistics

The VCF files of variant gene loci were statistically analyzed by self-established shell language, perl language and python tool, and the proportion of each variant site in different groups was calculated by AD information.

Hierarchical clustering analysis was performed using the hclust package in R language. The average aggregation clustering strategy was selected for hierarchical clustering of the distance metrics between strains calculated above, and the median unweighted pairwise group method (UPGMA) was used as the average clustering method to generate the clustering results. RandomForest package was used to predict high contribution gene sets.

### 2.10. Statistical Analysis

The data are expressed as means ± SD. Statistical significance was determined based on one-way analysis of variance (ANOVA) in appropriate conditions using GraphPad Prism 8 software. Significance was determined at *p* < 0.05.

## 3. Results

### 3.1. General Genomics Features of SP206-2

**Figure 1:**
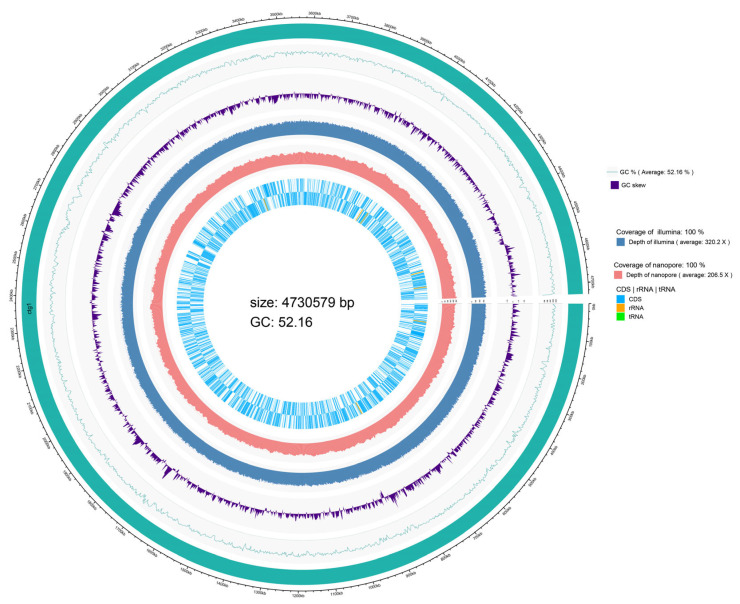
Complete genome of the arthritic *Salmonella* pullorum isolate SP206-2. The first circle from outside to inside is the genome sequence information. In the second circle, the GC content curve of the genomic sequence was sliding across the genome with a sliding window of 2000 bp, and the average GC content was calculated. The dashed line shows the average GC content of the reference genome. In the third circle, the GC skew curve of the genomic sequence was slid across the genome with a sliding window of 2000 bp, and the average GC content was calculated. The dashed line shows the reference line with GC skew 0. In the fourth circle, the depth and coverage information of next-generation sequencing were slid on the genome with 2000 bp as a sliding window, and the average sequencing depth was calculated to reflect the coverage of reads in different regions. The dashed line shows the average reads coverage at the overall level. In the fifth circle, the third-generation sequencing depth and coverage information were slid on the genome with 2000 bp as a sliding window, and the average sequencing depth was calculated to reflect the coverage of reads in different regions. The dashed line shows the average reads coverage at the overall level. The sixth circle shows the CDs and non-coding RNA regions (rRNA, tRNA) in the reference genome, which are represented by two inner and outer layers, the outer layer represents the plus strand, and the inner layer represents the minus strand.

The complete genome sequence of the arthritis-causing *S.* pullorum strain SP206-2 was determined and annotated (Table S1). The draft genome of SP206-2 was composed of 4,730,579 bp with G + C content of 52.16%, 5007 predicted genes (including 4729 CDSs), and 1426 pseudogenes (Figure 1, Table 2). The databases UniProt, KEGG, KEGG Pathway, GO, Pfam, COG, TIGERfams, RefSeq, and NR were used to annotate 4106, 1707, 1645, 3770, 4366, 2313, 2820, 4691, and 4007 genes, accounting for 82.01%, 34.09%, 32.85%, 75.29%, 87.20%, 46.20%, 56.32%, 93.69%, and 80.03% of the total genes number, respectively (Figure S1). Additionally, 4277 resistance genes were annotated using the CARD database.

### 3.2. Genomic Variability Analysis of Arthritis-Causing S. pullorum

**Figure 2:**
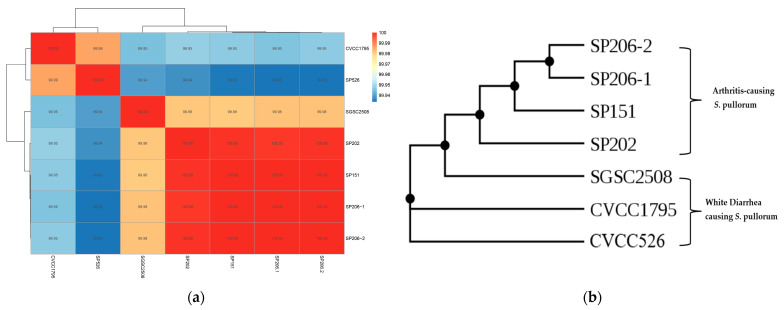
(**a**) Heat map of ANI analysis of *S*. pullorum. The depth of the color represents the value of the similarity. (**b**) Phylogenetic tree-based SNP.

Four sequenced arthritis-causing *S*. pullorum were compared with CVCC526, CVCC1795 (GCF_019990585.1), and SGSC25088 (GCA_018338655.1) in variant analysis of genomics. ANI is an important measure for determining the genetic association between species; it analyzes and compares homologous gene sequences based on the whole genome sequence of species. The results revealed that genetic similarity between arthritis-causing *S.* pullorum and diarrhea-causing *S.* pullorum was more than 99%, and the similarity among arthritis-causing *S.* pullorum strains was higher (Figure 2a).

SNP involves DNA sequence polymorphism caused by single nucleotide variation at the genomic level; it includes single base conversion, transversion, and the like. The SNP analysis-based phylogenetic tree revealed that the arthritis-causing *S.* pullorum strains and the diarrhea-causing *S.* pullorum strains belong to different branches, as shown in Figure 2b.

### 3.3. Gene Variations between Arthritis-Causing and Diarrhea-Causing S. pullorum

The genome of SP206-2 was used as a reference to discover the differentially expressed genes between arthritis-causing and diarrhea-causing *S.* pullorum. Twenty-six genes were found to be differentially among the different pathogeneses of *S.* pullorum (Figure S2), and an additional eight unannotated genes (hypothetical protein) showed differences. Further analysis of the enriched differential genes revealed ten protein-coding genes with significant mutation, which resulted in the deletion of encoded amino acids or the termination of translation, which in turn affected function (Table 3). The major differences, along with the genes in each category, are synthesis of substances (*thrL*, *guaB*, *treZ*, and *ccmF-1*), electron transport in the respiratory chain (*qorB*), virulence-related effector proteins (*tagO* and *sptP*), energy metabolism (*hpaI* and *rbsC*), and drug resistance gene (*mdtH*). *ThrL*, *guaB*, *treZ*, and *ccmF-1* are associated with the synthesis of threonine, guanine nucleoside, trehalose, and cytochrome C, respectively. The protein encoded by *qorB* is associated with electron transport in the respiratory chain. The proteins encoded by *tagO* and *sptP* are related to the type Ⅵ secretion system effector TagO and the type Ⅲ secretion system effector SptP, respectively. The proteins encoded by *hpaI* and *rbsC* are associated with pyruvate production and ATP energy metabolism. The *mdtH*-encoded protein mediates the norfloxacin resistance phase.

### 3.4. Relative Expression of Differentially Expressed Genes

**Figure 3:**
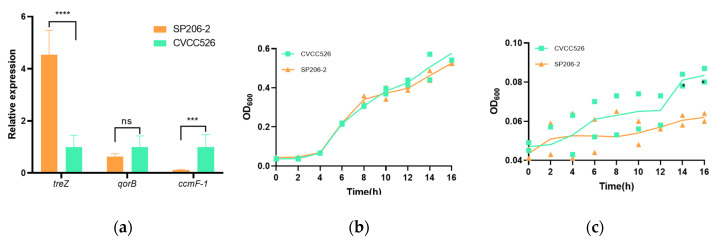
(**a**) Relative expression of *treZ*, *qorB*, and *ccmF-1* in SP206-2 and CVCC526. (**b**) Growth curves of SP206-2 and CVCC526 in LB broth medium. (**c**) Growth curves of SP206-2 and CVCC526 in M9 medium. *, *p* < 0.05 (One-way ANOVA); ***, *p* < 0.001; ****, *p*< 0.0001; ns, no significance.

Differences in the expression of *treZ*, *qorB*, and *ccmf-1* were determined using qPCR analysis. As shown in Figure 3a, the expression of *treZ* was identified to be significantly upregulated (>4-fold) compared with CVCC526 (*p* < 0.05). Although the relative expression of *qorB* was lower in SP206-2, there was no significant difference in fold changes. However, the expression of *ccmF-1* in SP206-2 was nearly 10-fold lower than that in CVCC526 (*p* < 0.05).

### 3.5. Growth Differences between Arthritis-Causing and Diarrhea-Causing S. pullorum

To determine differences in growth between SP206-2 and CVCC526 in various nutrient environments, the growth statuses of two *S.* pullorum strains in nutrient-sufficient LB broth medium and nutrient-poor M9 medium were compared. As shown in Figure 3b, before 10 h, the growth curves of SP206 and CVCC526 in the LB medium essentially coincided, and CVCC526 showed a slight growth advantage after 10 h. While both strains grew slowly in the M9 medium, CVCC526 showed obvious advantages in growth compared to SP206-2 (Figure 3c).

### 3.6. Drug-Resistant Phenotypic Differences of S. pullorum Strains

**Figure 4:**
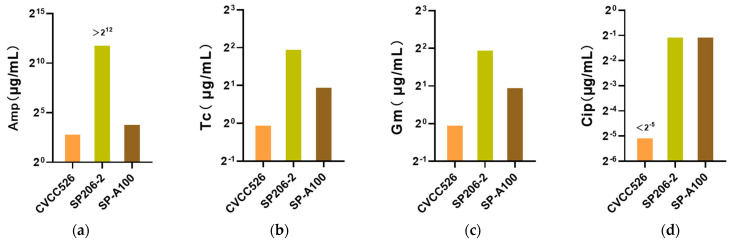
(**a**) MIC of ampicillin against CVCC526, SP206-2 and SP-A100. (**b**) MIC of tetracycline against CVCC526, SP206-2, and SP-A100. (**c**) MIC of gentamicin against CVCC526, SP206-2, and SP-A100. (**d**) MIC of ciprofloxacin against CVCC526, SP206-2, and SP-A100.

The MICs of antibiotics against different pathogenic *S.* pullorum strains were determined using the broth microdilution method. As shown in Figure 4, according to the test results, the MIC of β-lactam antibiotic ampicillin against CVCC526 was 8 µg/mL, but the MIC against SP206-2 was 4096 µg/mL and above. Tetracycline inhibited the growth of CVCC526 at a concentration of 0.5 µg/mL. Although the growth of SP206-2 was inhibited at 2 µg/mL, both strains were sensitive to tetracycline under the CLSI protocol. CVCC526 was sensitive to quinolone antibiotic ciprofloxacin, and its growth was inhibited when the concentration was less than 0.03 µg/mL. In contrast to CVCC526, ciprofloxacin exhibited an MIC of 0.25 µg/mL against SP206-2. In terms of aminoglycoside antibiotics, both strains were susceptible to gentamicin. In conclusion, the arthritis-causing *S.* pullorum SP206-2 were more resistant to antibiotics than CVCC526. Although the antibiotic resistance of SP-A100 was higher than that of CVCC526, it was still lower than that of SP206-2.

## 4. Discussion

*S.* pullorum primarily infects chicks, causing acute and systemic infection with high mortality. The majority of adult chickens develop latent and persistent infections; *S*. pullorum mainly attacks the reproductive system, inducing diseases such as oophoritis and salpingitis, but presents no obvious lesions or clinical signs in appearance [25]. *S*. pullorum cannot be completely eliminated by conventional drug treatment due to the characteristics of intracellular parasites. Furthermore, it can survive in the macrophages of the liver and spleen for a long time, resulting in persistent infection and long-term intermittent shedding. Therefore, infected chickens are a critical source of infection for the spread of *S.* pullorum [26].

Movement disorders induced by leg joint injuries remain a huge challenge for the poultry industry. Not only do they cause serious economic losses, but they also concern the welfare of chickens. Nowadays, more and more cases of chicken arthritis caused by *S*. pullorum infection have been reported, which has become a new epidemic feature of *S.* pullorum in China [7]. In this study, four strains of *S.* pullorum were isolated from the joint fluid, livers, and spleens of native Chinese Qingjiaoma chickens with clinical signs of tarsal joint swelling and arthritis. The pathogenic characteristics of the arthritis-causing *S.* pullorum strain SP206-2 were investigated in our earlier studies as well. The results also indicated that besides high virulence, SP206-2 also had a strong negative effect on the growth performance of chickens. It is worth noting that lameness and arthritis occurred 10 days after infection, with the incidence of arthritis at 27.78% (5/18). In addition, SP206-2 has been re-isolated from the synovial fluid and organs of dead chicks with arthritis clinical signs [19].

Pseudogenes are the alleles of a “normal” gene that has lost its function of encoding protein as a result of an accumulation of mutations. They are commonly observed in *Salmonella* genomes and are usually created by deletions or insertions that cause a frame shift or by a nonsense SNP that result in a stop codon in coding region, which reflected adaptation to the changing environment [27]. Previous research has shown that the abundance of pseudogenes in *S*. pullorum is the result of host adaptation [27], and statistics have shown that the *S*. enteritidis strain P125109 has only 48 pseudogenes, while the strains *S*. pullorum RKS5078 and *S*. Gallinarum 287/91 had as many as 263 and 215 pseudogenes, respectively [18]. The number of pseudogenes of the strains CVCC1795 (GCF_019990585.1) and SGSC2508 (GCA_018338655.1) used in this study were 456 and 308, respectively; however, the arthritic *S.* pullorum strain SP206-2 had 1426 pseudogenes, indicating that the genome of this strain has been more seriously degraded. This may be the result of the adaptation of SP206-2 to the joint environment, contributing to better colonization in the joint.

Comparative genomics is the method of sequencing the whole genome of the individuals of a species and analyzing the differences in information [28]. The ANI calculation makes use of a large number of genes; in contrast to 16S rRNA, it is not affected by the inheritance rate and the horizontal gene transfer of a single gene or few genes. Even though a large proportion of genes in the genomes of species have different evolutionary histories, the distribution of ANI values of subspecies is 90–96%. Although the ANI between arthritis-causing strains and white diarrhea-causing strains was more than 99%, the similarity among the genomes of arthritis-causing strains was higher, which means the genomic characteristics of arthritis-causing *S.* pullorum strains can be distinguished from those of white diarrhea-causing *S.* pullorum strains.

The enrichment of genes with SNP and Indel non-synonymous mutations in this study revealed that the mutated genes were mainly related to substance synthesis, electron transport in the respiratory chain, virulence-related effector proteins, energy metabolism, and drug resistance genes, indicating that the arthritis-causing strains have adaptive changes in order to live in joint fluid. One of these mutant genes, *treZ*, encodes a malto-oligosyltrehalose trehalohydrolase that can catalyze the synthesis of trehalose in synergy with oligosyltrehalose trehalosynthetase. Extant research has suggested that *E. coli* strains have attained hyperosmotic pressure resistance, to withstand high-salt environments through the accumulation of intracellular trehalose [29]. Using infrared spectroscopy to analyze the intracellular trehalose in lactic acid bacteria during freeze-drying, Zhang et al. found that trehalose formed hydrogen bonds with the components of cell membranes to replace water to maintain the cell membrane [30]. In our study, the expression of *treZ* in the SP206-2 strain was significantly higher than that in CVCC526, indicating that the *treZ* mutation in the arthritis-causing *S.* pullorum may well be related to survival in the hypertonic environment of arthritis. *QorB* encodes a quinone oxidoreductase, the first enzyme in the bacterial respiratory chain that oxidizes NADH (reduced coenzyme I) to NAD+ and produces protons for ATP synthesis. A previous study has shown that overexpression of the gene encoding quinone oxidoreductase in *E. coli* results in growth retardation of the strain and a significant reduction in the content of enzymes related to glucose metabolism [31]. In our study, the growth trends of SP206-2 and CVCC526 were not significantly different from each other in the nutrient-sufficient LB medium; however, the growth of SP206-2 was significantly slower than that of CVCC526 in the nutrient-poor M9 medium, further confirming the lack of metabolism-related enzymes in SP206-2. *Ccmf-1* encodes a protein involved in cytochrome C synthesis, CcmF, which catalyzes the formation of mature cytochrome C from heme and cytochrome C precursor proteins. The qPCR results showed that the expression of *ccmF-1* was significantly down-regulated in SP206-2. Cytochrome C is a vital electron carrier in the respiratory chain, transferring electrons between cytochrome C reductase and cytochrome C oxidase through changes in the valence of iron ions in heme [32]. Two important proteins concerned with the respiratory chain in arthritis-causing *S.* pullorum were mutated, indicating that the energy metabolism of *S.* pullorum may have been changed.

According to the MIC results, both SP206-2 and SP-A100 were more resistant to antibiotics than CVCC526. This is relevant to the transfer of drug-resistant during cultivation. Moreover, SP206-2 showed stronger resistance to the selected antibiotics than SP-A100, indicating that there may be more resistance genes in arthritis-causing *S.* pullorum, which presents greater challenge to its prevention and control. With antibiotic resistance being a serious threat to veterinary and public health, the prudent use of antibiotics receives much attention. Less well known is that incorrect use of antimicrobial agents such as tetracycline may also lead to increased bacterial virulence with the potential of a more severe clinical course of infection [33]. Under strong virulence, the SP206-2 strain has been found. This study has also shown that preadaptation to tetracycline or ciprofloxacin can lead to cross-resistance to antibiotics, which is relatively consistent with our results [34]. These data further highlight the importance of prudent use of antibiotics.

## 5. Conclusions

In summary, through comparative genomics analysis, we found that the genome of the arthritis-causing *S.* pullorum has undergone further degradation compared to that of white diarrhea-causing *S.* pullorum. The number of pseudogenes increased by more than threefold, and many genes related to substance synthesis and energy metabolism were mutated, indicating that the gene level of the arthritis-causing *S.* pullorum strains had undergone adaptive changes. These data fill a gap in the genomics of arthritis-causing *S*. pullorum and lays a foundation for understanding the pathogenic diversity and the purification of *S.* pullorum in chickens. As the arthritis-causing *S*. pullorum from only one farm and no information on the arthritic *S*. pullorum was found on the website, our conclusions only support the strains we isolated.

## Figures and Tables

**Table 1:** Mutant genes qPCR primer sequence.

Gene	Primer Sequences	Fragment Size (bp)	Annotation
*treZ*-L *treZ*-R	cgatccggcttcccgcgcccccagccacgctcgccgccaa	259	Trehalose hydrolase
*qorB*-L *qorB*-R	agcgcgcattgcggaggcgagcaccgcgcagcaccgcaac	278	NAD(P)H dehydrogenase
*ccmF-1*-L *ccmF-1*-R	gcgggcgcgggagccgtatcggcgcgcccaccgaaagcct	129	Cytochrome C synthetase
16S rRNA-L 16S rRNA-R	cagggggccgccttcgccacagcgcacgcaggcggtctgt	168	Reference genome

**Table 2:** Prediction results of genes.

Type	Number	Total Length (bp)	Average Length (bp)
Gene	5007	4,306,080	860
CDS	4729	4,240,824	897
tRNA	75	5907	79
23S rRNA	7	20,909	2987
16S rRNA	7	10,710	1530
5S rRNA	8	920	115
tmRNA	1	363	363
Misc RNA	180	26,447	147
Pesudogene	1426	391,080	274.25

**Table 3:** Differential gene mutation types and encoded protein functions.

Gene	Mutation Type	Mutation Site	Amino Acid Mutation	Encoded Protein
*mdtH*	Disruptive inframe deletion	656~673-bit deletion GCTACTATATGCTGGCGG	Gly219~Ala224 deletion	Norfloxacin resistant protein MdtH
*tagO*	Conservative inframe deletion	469~507 -bit deletion CACGCCATGACGCCGGACAGCATCGACGTGACGCTGACC	His157~Thr169 deletion	Type VI secretory system associated proteins TagO
*rbsC*	Disruptive inframe deletion	264~308-bit deletion TTCAATCGTCGGCGTTGAAGTGAATGCGCTGGTCGCGGTTGCCGC	Ser89~Ala103 deletion	Ribose transporter RbsC
*sptP*	Frameshift variant & stop gained	369~370 sequence deletion TGTTGCGCCTGAAAAATTTTCGTCAAAAGTATTAACCTGGCTTGGAAAAATGCCGTTATTTAAAAACACTG	Lys124 termination of translation	Secretory effector protein SptP
*thrL*	Disruptive inframe deletion	33~47-bit deletion CATCACCATTACCAC	Ile12~Thr16 deletion	Threonine operon precursor peptide
*guaB*	Conservative inframe deletion	1447~1449-bit deletion AAC	Asn483 deletion	Inosine monophosphate dehydrogenase
*hpaI*	Frameshift variant	362~378-bit deletion TGCTGGCGCGGGCATCG	Val121 termination of translation	P-hydroxyphenylacetic aldolase
*ccmF-1*	Disruptive inframe insertion	1039~1040sequence deletion GTTTGGGCGGCGGAAAGTGCGAAACGCTCATTCTGGCGACGC	Thr346~Leu347 deletion	Cytochrome C synthetase CcmF subunit
*qorB*	Frameshift variant	648~649-bit deletion AA	Ser217 termination of translation	Quinone oxidoreductase
*treZ*	Frameshift variant	3~4-bit deletion TT	Val2 termination of translation	mahosyhrehalose hydrolase

## Data Availability

The Salmonella WGS data have been deposited to NCBl (CVCC526 Bioproject accession number: PRJNA1035463) (SP206-2 Bioproject accession number: PRJNA1035419).

## Supplementary Materials

The following supporting information can be downloaded at: https://www.mdpi.com/article/10.3390/microorganisms11122986/s1, Table S1. Sequencing data quality assessment. Table S2. Drug-resistant phenotypes of *Salmonella* pullorum isolates. Figure S1. Statistical chart of annotations common and unique to the universal database of coding gen. Figure S2. Differential genes between diarrhea-causing *S*. pullorum and arthritic-causing *S*. pullorum.
