# Supplementary material for: The Genomic Characteristics of an Arthritis-Causing Salmonella pullorum
Source: Microorganisms. 2023 Dec 14;11(12):2986. doi: 10.3390/microorganisms11122986 (PMC10745331; doi:10.3390/microorganisms11122986)
Supplement: Supplementary file 1 [file microorganisms-11-02986-s001.zip › microorganisms-2751470-supplementary.pdf]

Supplementary Materials: Raw Reads were obtained by sequencing data, and high-quality Clean Reads were obtained after filtering and quality control. The Q20 quality control rate of each isolate was above 97%, and the Q30 quality control rate was above 93%, indicating that the obtained Clean Reads had high quality and high reliability.

Supplement Table S1. Sequencing data quality assessment.

**Strains**	**Raw Reads**	**Clean Reads**	**Q20**	**Q30 [30]**	**GC Content [30]**
SP151	4,417,273	4,413,385	97.97	93.84	52.25
SP202	5,057,985	5,054,044	98.11	94.18	52.89
SP206-1	4,479,104	4,475,582	98.10	94.17	52.35
SP206-2	4,276,605	4,271,758	98.15	94.31	52.44

Supplement Table S2. Drug-resistant phenotypes of Salmonella Pullorum isolates

Isolate	Antimicrobial resistance pattern
SP151	AMP-AMX-NAL
SP202	AMP-AMX-NAL
SP206-1	AMP-AMX-STR-NAL
SP206-2	AMP-AMX-STR-NAL

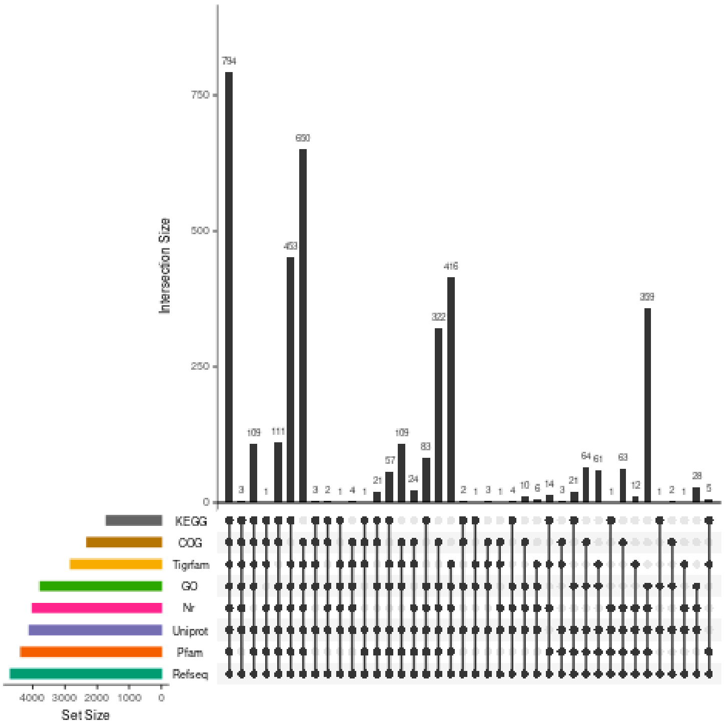

Supplement Figure S1. Statistical chart of annotations common and unique to the universal database of coding gen. The databases UniProt, KEGG, KEGG Pathway, GO, Pfam, COG, TIGERfams, RefSeq, and NR were used to annotate 4106, 1707, 1645, 3770, 4366, 2313, 2820, 4691, and 4007 genes, accounting for 82.01%, 34.09%, 32.85%, 75.29%, 87.20%, 46.20%, 56.32%, 93.69%, and 80.03% of the total gene number, respectively; 4277 resistance genes were annotated using the CARD database.

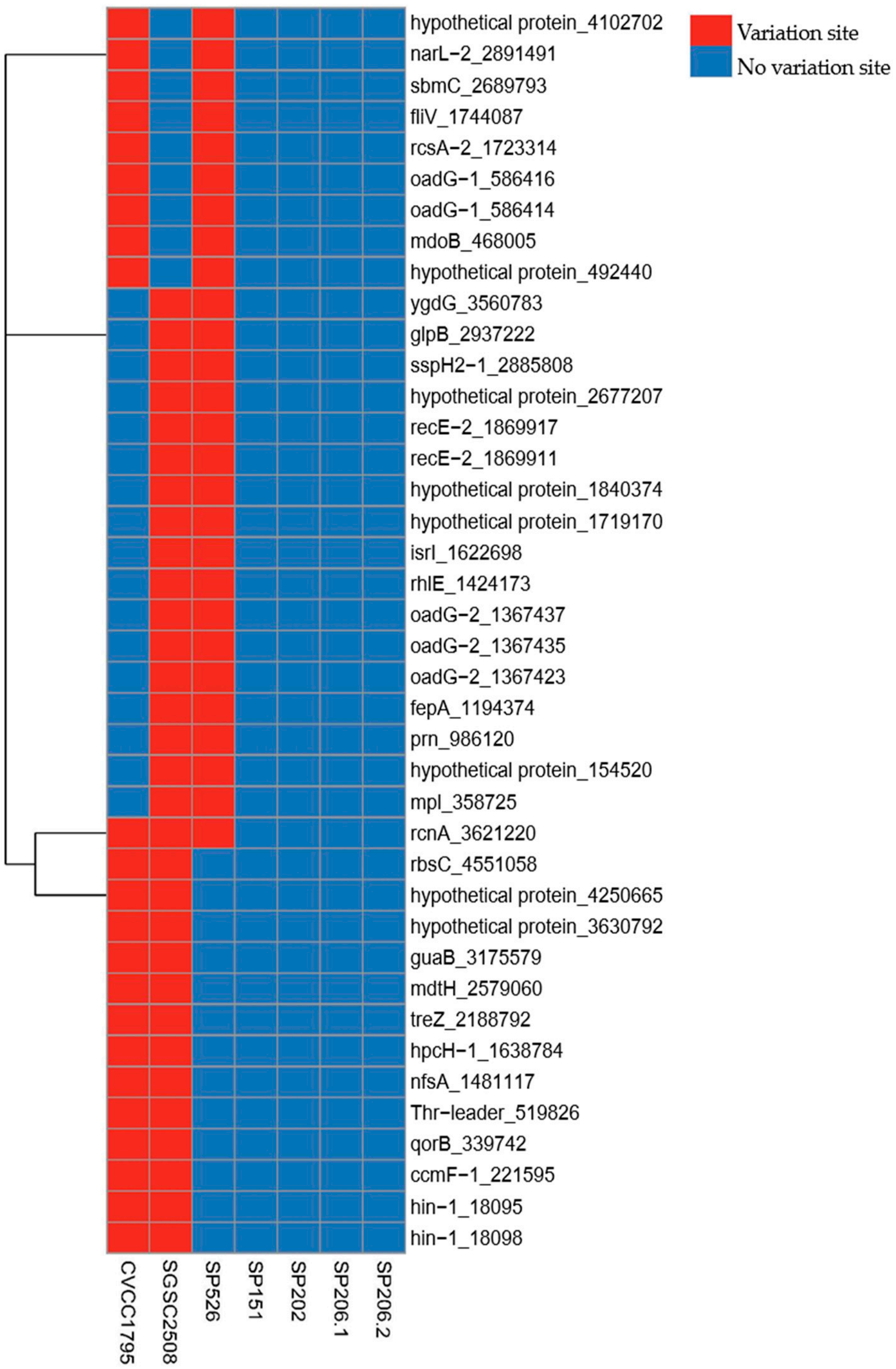

Supplement Figure S2. Differential genes between diarrhea-causing S. Pullorum and arthritic-causing S. Pullorum.
